# Efficacy of human prothrombin complex concentrate in the treatment of warfarin overdose in patients receiving warfarin for mechanical heart valve replacement

**DOI:** 10.1097/MD.0000000000038022

**Published:** 2024-05-10

**Authors:** Hasan Uncu, Tolga Onur Badak, Haci Ali Ucak, Ferid Cereb, Ahmet Cakallioglu

**Affiliations:** a Adana City Training and Research Hospital Department of Cardiovascular Surgery, Adana, Turkey; b Adana Cukurova State Hospital Department of Cardiovascular Surgery, Adana, Turkey.

**Keywords:** anticoagulation, mechanical heart valve, prothrombin complex concentrate, reaching target INR, warfarin overdose

## Abstract

Warfarin, a widely utilized anticoagulant, is paramount for preventing thromboembolic events in patients with mechanical heart valve replacements. However, its narrow therapeutic index can lead to over-anticoagulation and overdose, resulting in serious health risks. This study examines the efficacy of human prothrombin complex concentrate (PCC) in managing warfarin overdose, in comparison with traditional treatments. A retrospective analysis was conducted on 162 adults who presented with warfarin overdose (INR > 5.0) at a tertiary care hospital between 2016 and 2020. Participants were divided into 2 groups—those treated with PCC (n = 57) and those treated with conventional methods (n = 105), including vitamin K and fresh frozen plasma. The primary outcome was the rate of reaching the target (International Normalized Ratio) INR within 24 hours. Secondary outcomes included transfusion requirements, thromboembolic events, adverse reactions, 30-day mortality, and length of hospital stay. PCC demonstrated significant efficacy, with 89.5% of patients achieving the target INR within 24 hours, compared to 64.8% in the control group (*P* < .05). The PCC group also had reduced transfusion requirements and a shorter average hospital stay. There was no significant difference in thromboembolic events or adverse reactions between the 2 groups, and the reduced 30-day mortality in the PCC group was not statistically significant. Human prothrombin complex concentrate is associated with rapid reaching the target INR, decreased transfusion needs, and shortened hospitalization, making it a promising option for warfarin overdose management. While the results are encouraging, larger, multicenter, randomized controlled trials are necessary to further validate these findings and optimize PCC administration protocols.

## 1. Introduction

Warfarin, an anticoagulant widely prescribed to prevent thromboembolic events, has been a cornerstone of medical treatment for patients with various cardiac and vascular disorders for decades.^[1]^ Patients undergoing heart valve replacement require particularly careful anticoagulation due to the increased risk of thromboembolic events. In this context, warfarin stands out with its proven efficacy and safety. A study by Altiok showed that the use of warfarin significantly reduced thromboembolic events in this patient population.^[2]^ Studies on alternative direct oral anticoagulants are limited in providing adequate safety and efficacy in patients with heart valve replacement.^[3]^ This confirms that warfarin is still the most safe and effective anticoagulation strategy for this patient group. While effective in its therapeutic role, the narrow therapeutic index of warfarin poses a significant challenge for healthcare providers, as slight deviations from the therapeutic range can lead to both under-anticoagulation or over-anticoagulation.

Warfarin overdose, though infrequent, can have serious or even fatal consequences, including uncontrolled hemorrhage.^[4]^ The management of warfarin overdose requires prompt intervention and careful monitoring, often in a critical care setting. Traditionally, the treatment includes the administration of vitamin K, fresh frozen plasma, or other clotting factors.^[5]^

In recent years, PCC has emerged as an alternative treatment option in the reversal of warfarin overdose. PCC has been shown to have certain advantages over traditional treatment methods, such as rapid onset of action and reduced volume overload.^[6]^ However, data on the efficacy and safety of PCC in the clinical management of warfarin overdose are still evolving.

This retrospective study aims to explore the efficacy of human prothrombin complex concentrate in the management of warfarin overdose, comparing the outcomes and safety profile with traditional treatment methods. Such a study could contribute to the evolving body of knowledge on warfarin reversal strategies, offering potentially more effective, rapid, and patient-tailored approaches to care.

## 2. Methods

### 2.1. Study design and setting

This retrospective study was conducted at a large tertiary care hospital, encompassing data from January 2016 to December 2020. The study design and protocol were approved by the Institutional Review Board (IRB), ensuring compliance with ethical guidelines outlined in the Declaration of Helsinki.

### 2.2. Participants

#### 2.2.1. Selection criteria

Patients eligible for this study were adults (age ≥ 18 years) who presented to the hospital with a confirmed diagnosis of warfarin overdose, characterized by an INR >5.0. The confirmation of warfarin overdose was made through laboratory testing, and a thorough review of the patient medical and medication history.

#### 2.2.2. Exclusion criteria

The study excluded patients who had contraindications to PCC, such as known allergies to any components of the concentrate. Additionally, individuals with underlying clotting disorders that could confound the results, patients with incomplete medical records, or those who received other nonstandard treatments for warfarin overdose were also excluded.

#### 2.2.3. Study cohort

The cohort comprised 2 well-matched groups:

1. PCC group included 57 patients who were treated with human prothrombin complex concentrate for warfarin overdose.
2. Control group: A comparable number of patients treated with traditional methods for warfarin overdose, such as vitamin K and fresh frozen plasma, were included in this group.

#### 2.2.4. PCC group intervention

##### Treatment protocol:

Patients in the PCC group received human prothrombin complex concentrate, as guided by recent recommendations.^[7]^ The dose was adjusted according to the patient body weight and initial INR, to tailor the treatment for individual needs.

Continuous monitoring of coagulation parameters was performed to assess the response to treatment and to ensure timely adjustments if necessary.

Follow-up: Short-term follow-up was conducted to evaluate any immediate adverse reactions or complications related to the treatment.

#### 2.2.5. Control group intervention

##### Treatment protocol:

Patients in the control group received conventional treatment with vitamin K and/or fresh frozen plasma, as outlined in the American College of Chest Physicians guidelines.^[8]^ The selection of treatment was based on the severity of overdose, patient clinical condition, and the physician judgment.

Similar to the PCC group, continuous monitoring was performed to track the patient response to treatment. Follow-up care was provided to evaluate the efficacy of the treatment and monitor for potential complications or side effects.

Treatment Team: Both intervention groups were managed by a specialized cardiovascular surgery team including critical care expert of cardiac surgery critical care unit. The team had expertise in managing warfarin overdose and worked in close collaboration to provide individualized care. Regular meetings were held to discuss patient progress, and any challenges or clinical decisions were made collectively.

### 2.3. Data collection

Data were extracted from electronic health records and included demographic information, medical history, initial INR, treatment details, time to reach the target INR, complications, and hospitalization length. A standardized form was used to ensure consistency in data collection.

### 2.4. Outcome measures

The primary outcome was the efficacy of human PCC in reaching the target INR within 24 hours. Secondary outcomes included transfusion requirements, thromboembolic events, adverse reactions to treatment, and overall mortality within 30 days.

### 2.5. Statistical analysis

Data were analyzed using SPSS version 26.0 (IBM Corp., Armonk, NY). Continuous variables were compared using the Student *t* test or the Mann–Whitney U test, as appropriate. Categorical variables were analyzed using the chi-squared or Fisher exact test. A *P* value < .05 was considered statistically significant.

## 3. Results

### 3.1. Patient characteristics

A total of 162 patients were included in the study, divided into 2 groups: the PCC group (n = 57) and the control group (n = 105). The baseline characteristics, including age, sex, initial INR, and comorbidities, were comparable between the 2 groups, ensuring a balanced comparison (Table 1).

**Table 1:** Baseline characteristics of the study cohort.

Characteristics	PCC group (n = 57)	Control group (n = 105)
Age (yr), mean ± SD	68.4 ± 12.3	67.9 ± 13.1
Male, n (%)	32 (56.1%)	58 (55.2%)
Initial INR, mean ± SD	8.2 ± 1.1	8.3 ± 1.0
Comorbidities:		
- Hypertension, n (%)	20 (35.1%)	37 (35.2%)
- Diabetes, n (%)	15 (26.3%)	30 (28.6%)
- Coronary artery disease, n (%)	18 (31.6%)	33 (31.4%)
Prior bleeding, n (%)	12 (21.1%)	22 (21.0%)
Antiplatelets, n (%)	10 (17.5%)	19 (18.1%)

INR = international normalized ratio.

### 3.2. Primary outcome

Reaching the target INR within 24 hours PCC Group: 51 out of 57 patients (89.5%) achieved the target INR within 24 hours.

Control Group: 68 out of 105 patients (64.8%) achieved the target INR within the same timeframe.

The difference was statistically significant (*P* < .05), indicating that human prothrombin complex concentrate was more effective in achieving rapid reaching of the target INR (Table 2).

**Table 2:** Primary and secondary outcomes.

Outcomes	PCC group (n = 57)	Control group (n = 105)	*P* value
INR normalization within 24h, n (%)	51 (89.5%)	68 (64.8%)	<.05
Transfusion Requirements:			
- FFP, n (%)	6 (10.5%)	25 (23.8%)	<.05
- Platelets, n (%)	4 (7.0%)	15 (14.3%)	<.05
Thromboembolic events, n (%)	3 (5.3%)	6 (5.7%)	.27
Adverse reactions, n (%)	2 (3.5%)	4 (3.8%)	.56
30-d mortality, n (%)	4 (7.0%)	10 (9.5%)	.09
Length of ICU stay (d), mean ± SD	2.1 ± 0.8	3.4 ± 1.2	<.01

FFP = fresh frozen plasma, ICU = intensive care unit, INR = international normalized ratio.

### 3.3. Secondary outcomes

The PCC group required fewer blood product transfusions compared to the control group (*P* < .01) (Table 2).

No significant difference was observed in the occurrence of thromboembolic events between the 2 groups (*P* = .27).

Adverse reactions were minimal in both groups, with no significant difference detected (*P* = .56).

30-day Mortality: The overall mortality within 30 days was lower in the PCC group, but the difference did not reach statistical significance (*P* = .09).

The average length of hospitalization was shorter in the PCC group, at 4.2 ± 1.6 days, compared to 6.3 ± 2.1 days in the control group (*P* < .01) (Table 3).

**Table 3:** Hospitalization length and follow-up.

	PCC Group (n = 57)	Control Group (n = 105)
Hospitalization d, mean ± SD	4.2 ± 1.6	6.3 ± 2.1
Readmission within 30 d, n (%)	3 (5.3%)	7 (6.7%)
Complications at 30-d follow-up, n (%)	5 (8.8%)	12 (11.4%)

The results of this retrospective study demonstrate that the use of human prothrombin complex concentrate in the management of warfarin overdose was associated with quicker to reach the target INR, reduced transfusion requirements, and shorter hospitalization length when compared to traditional methods. The findings support the potential benefits of human PCC in the management of warfarin overdose, warranting further prospective research to confirm this result.

## 4. Discussion

The present retrospective study investigated the efficacy of human prothrombin complex concentrate in the management of warfarin overdose, encompassing 162 patients over a 5-year period. The findings demonstrate the advantages of human PCC in to rapid reaching the target INR, reduced transfusion needs, and shortened hospital stays compared to traditional treatments like vitamin K and fresh frozen plasma.

Warfarin overdose is a major medication complication that can lead to serious morbidity and mortality. Warfarin may interact with certain medications and foods and is responsive to gene polymorphism, consequently, it is often difficult to achieve the desired INR level. Even minor manipulations in medication use can sometimes lead to major INR changes. Low patient compliance is also an important reason for medication misuse. It is unknown what causes the majority of warfarin overdoses, but about half of patients with an INR of 10 or higher were found to abuse the medication.^[9]^ Warfarin overdose can lead to bleeding complications of varying severity depending on the INR level and the patient comorbidities. The severity of bleeding varies, ranging from epistaxis to intracerebral hemorrhage.^[10]^

Our results align with the current understanding of PCC role in reversing anticoagulation. A review by Sarode et al (2013) emphasized the superiority of PCC in promptly correcting INR compared to fresh frozen plasma, particularly in the context of urgent reversal requirements.^[11]^ The timely correction of INR is vital, especially in life-threatening situations, to minimize the risk of hemorrhage. İn our retrospective study, we acknowledge that patients with supra-therapeutic INR levels are at a heightened risk of active bleeding, and the treatment approach for such patients typically necessitates a more intensive and rapid reversal of anticoagulation. While our study does not specifically stratify patients based on the presence of active bleeding, it is possible that in clinical practice, patients with both supra-therapeutic INR and active bleeding were preferentially treated with human PCC due to its potential advantages in achieving rapid INR normalization.

The decision to administer PCC or conventional treatments, such as vitamin K and/or fresh frozen plasma, was based on several factors, including the severity of the overdose, the patient clinical condition, and the judgment of the treating physician or healthcare team. In cases of active bleeding or a high risk of bleeding complications, the choice of treatment may lean towards PCC as it offers a more targeted and immediate correction of coagulation parameters.

It is important to emphasize that our study reflects real-world clinical practice, where treatment decisions are made on a case-by-case basis to ensure the safety and well-being of patients. While we did not explicitly analyze the treatment preferences based on the presence of active bleeding, we acknowledge that this aspect could introduce variability in the choice of treatment modality.

The reduced need for transfusions in the PCC group is consistent with previous research, reflecting the higher concentration of clotting factors in PCC.^[12]^ This concentration allows for a more targeted and effective treatment, minimizing exposure to blood products and potential complications such as transfusion reactions or volume overload. While the difference in 30-day mortality was not statistically significant in our study, a trend toward lower mortality in the PCC group is noteworthy. Prospective trials with larger sample sizes may further elucidate this aspect, adding to evidence like that presented by Goldstein et al, where PCC was associated with improved survival rates in specific patient subgroups.^[13]^

Our study limitations include its retrospective nature and single-center design, which may introduce biases and limit the generalizability of the findings. Furthermore, the absence of a standardized protocol across both groups may have influenced the results. Future multicentre, randomized controlled trials would provide more robust evidence to guide clinical practice.

In conclusion, this study adds to the growing body of evidence supporting the use of human PCC in managing warfarin overdose. By demonstrating enhanced efficacy in reaching the target INR, decreased transfusion requirements, and potential improvements in overall patient outcomes, our findings align with recent guidelines advocating for PCC use in specific clinical scenarios.^[14]^ However, more extensive prospective research is warranted to confirm these results and to optimize the protocols for PCC administration in different clinical settings.

## Author contributions

**Conceptualization:** Hasan Uncu.

**Data curation:** Hasan Uncu, Tolga Onur Badak, Haci Ali Uçak.

**Formal analysis:** Hasan Uncu, Tolga Onur Badak, Haci Ali Uçak, Ahmet Cakallioglu.

**Funding acquisition:** Hasan Uncu, Haci Ali Uçak, Ferid Cereb.

**Investigation:** Hasan Uncu, Tolga Onur Badak, Haci Ali Uçak, Ferid Cereb, Ahmet Cakallioglu.

**Methodology:** Hasan Uncu, Tolga Onur Badak, Haci Ali Uçak, Ferid Cereb, Ahmet Cakallioglu.

**Project administration:** Hasan Uncu, Haci Ali Uçak, Ferid Cereb.

**Resources:** Hasan Uncu, Tolga Onur Badak, Ahmet Cakallioglu.

**Software:** Hasan Uncu, Tolga Onur Badak, Ferid Cereb, Ahmet Cakallioglu.

**Supervision:** Hasan Uncu, Tolga Onur Badak, Haci Ali Uçak, Ahmet Cakallioglu.

**Validation:** Hasan Uncu, Ferid Cereb.

**Visualization:** Hasan Uncu, Tolga Onur Badak, Haci Ali Uçak, Ferid Cereb.

**Writing – original draft:** Hasan Uncu.

**Writing – review & editing:** Hasan Uncu, Tolga Onur Badak, Haci Ali Uçak.
